# Inflammaging as a Dynamic Process in Ageing: Context, Regulation and Functional Consequences

**DOI:** 10.1098/rstb.2026.0155

**Published:** 2026-11-12

**Authors:** Muhammad Ali, Hafiz Muhammad Irfan, Kanwal Asif, Chandra Sekhar Bhol, Gautam Sethi, Vinay Tergaonkar, Aqeel Mahmood

**Affiliations:** ^1^ Department of Pharmacology, College of Pharmacy, University of Sargodha, 40100 Sargodha, Punjab, Pakistan; ^2^ Department of Pharmacology, Yong Loo Lin School of Medicine, National University of Singapore, 117600 Singapore; ^3^ Sydney Musculoskeletal Research Centre, Faculty of Medicine and Health, The University of Sydney, Sydney, Australia; ^4^ Laboratory of NF-κB Signalling, Institute of Molecular and Cell Biology (IMCB), Agency for Science, Technology and Research (A*STAR), 138673 Singapore; ^5^ Department of Biochemistry, Yong Loo Lin School of Medicine, National University of Singapore, 117596 Singapore; ^6^ FSCPO: Ministry of Education Frontiers Science Center for Precision Oncology, University of Macau, Taipa, Macao, People's Republic of China; ^7^ Cancer Center, Faculty of Health Sciences, University of Macau, Taipa, Macao, People's Republic of China; ^8^ Department of Pharmacy, Faculty of Health and Medical Sciences, Mirpur University of Science and Technology, 10224 Mirpur, Azad Jammu and Kashmir, Pakistan

**Keywords:** inflammaging, senescence cells heterogeneity, cancer–ageing paradox, senescence-associated secretory phenotype, temporal dynamics

## Abstract

Inflammaging (InfA) is a chronic, sterile, low-grade inflammatory condition associated with ageing and the development of age-related diseases (ARDs), including cancer. Conventional models often portray InfA as a relatively homogeneous and progressively worsening process, which may not adequately capture its temporal dynamics, tissue specificity, reversibility and context-dependent biological consequences. Here, we propose that InfA is better understood as a dynamic state-transition process comprising resolved/adaptive (S0), compensatory (S1), maladaptive but potentially reversible (S2) and self-sustaining (S3) inflammatory states. Transitions among these states are shaped by the balance between inflammatory induction and resolution, senescent cell (SC) generation and immune-mediated clearance, metabolic and mitochondrial resilience and microenvironmental feedback. We further integrate SC heterogeneity and immune surveillance into this framework, proposing that declining clearance and emerging immune escape permit persistent senescent phenotypes to establish self-reinforcing inflammatory microenvironments. This model also provides a mechanistic perspective on the cancer–ageing paradox by explaining how initially protective processes, including senescence, autophagy and immune surveillance, may be progressively re-tuned toward maladaptive functions during ageing. S2 emerges as a particularly important therapeutic window during which inflammatory dysregulation and immune escape are established but may remain conditionally reversible. Tissue-specific biomarkers may enable state- and timing-informed InfA interventions and provide a foundation for precision geroscience.

This article is part of the theme issue ‘Developmental circuits in ageing’.

## 1. Introduction: Ageing and chronic inflammation

One of the most significant demographic transformations of the 21st century is population ageing. The world is witnessing a rapid growth in the number of people aged 60 years and above, which has consequently led to a corresponding growth in the number of CNCDs across the world. It is estimated that by 2050, the population aged ≥60 years will exceed 2.1 billion, representing 21% of the world population [1]. Cancer, neurodegenerative diseases, metabolic disorders and cardiovascular diseases are disproportionately affecting the ageing population and contribute to the majority of global morbidity and mortality [2].

A common biological denominator among most ARDs is chronic low-grade systemic inflammation. There is a growing consensus that this chronic inflammatory environment is a major molecular cause of tissue dysfunction, organ loss and disease vulnerability rather than just a by-product of ageing. Understanding the mechanisms that produce and modulate this inflammatory condition has thus become a central focus of modern ageing research and geroscience [3].

Ageing progressively alters physiological systems through cumulative biological, metabolic and environmental stress owing to various biological, physical and chemical factors. Across the lifespan, the regulatory networks that sustain physiological stability are progressively remodelled by cumulative molecular damage, metabolic stress and environmental exposures [4]. The long-term consequence of this progressive process is systemic decline driven by increasing biological entropy and reduced physiological resilience. As resilience declines, tissues and organs experience chronic dysfunction because the capacity to maintain the homeostatic balance also weakens. InfA may represent one manifestation of this decline in systemic regulatory capacity [5].

The term ‘InfA’ was introduced by Franceschi and colleagues in 2000 and has evolved considerably as new evidence has emerged. It refers to a ‘sterile, non-resolving, chronic, low-grade inflammatory state’ that changes the expression of pro-inflammatory mediators [6]. InfA is not considered a sudden pathological transition but rather a gradual shift in homeostatic balance resulting from continuous remodelling of the immune system [7]. This view was strengthened by further discoveries in the field of geroscience, which linked InfA to immunosenescence—the age-related deterioration of innate and adaptive immunity. Immunosenescence hallmarks such as changes in CD4^+^/CD8^+^ T-cell ratios, loss of T-cell receptor diversity and loss of naive T-cell production are signs of loss of immune adaptability rather than homogeneous immune suppression [8]. Collectively, these modifications suggest that InfA is the outcome of the long-term interaction of immune control, metabolic state and tissue homeostasis and, therefore, it cannot be considered a unitary, homogeneous phenotype.

To improve the understanding of this complexity, several authors propose stage- or phase-based models of inflammation associated with ageing. One example is the concept of ‘cold inflammaging,’ which is thought to progress through a pre-inflammatory dyshomeostatic stage before progressing to chronic low-grade InfA. This process eventually results in the development of ARDs [9,10]. Even though the stage- or phase-based models proved successful in structuring the observations and making them easier to interpret, their broader biological generalization warrants careful consideration, particularly regarding how these models apply to various ARDs and the underlying mechanisms of InfA.

Much of the evidence supporting staged progression is obtained by cross-sectional comparisons of biomarkers and not by longitudinal follow-up of the same individuals. Inter-individual differences may reflect inter-individual variation rather than sequential changes across time [11]. Inflammatory activities associated with ageing are slow and, in most instances, non-linear with time. InfA has highly context-dependent biological effects, and similar immune signals can have varied effects based on physiological conditions, such as the presence of chronic diseases or the individual's overall health status [12].

Moreover, the inflammatory changes caused by ageing are highly tissue-specific. Such differences are signs of disparities in the immune system composition, energy requirements, regenerative capacity and the history of exposure between organs [13]. Consequently, InfA can be considered a heterogeneous and spatially diffused process. The pattern of inflammation varies across tissues over time and does not follow a coordinated series of rigid phases.

Further complexity emerges at the intersection of ageing and the most common ARD, i.e. cancer. This cancer–ageing paradox may seem disconnected, but it is a more complicated and interwoven process. The increased incidence of cancer in old age and the simultaneous presence of tumour-suppressive mechanisms in centenarians expose the fundamental conceptual tensions that are difficult to comprehend with linear frameworks of InfA [14].

In addition to these general considerations, certain biological phenomena complicate stage-based interpretations of InfA. The inflammatory cues released by SCs and their corresponding SASP are spatially heterogeneous and temporally dynamic [15]. Considering such observations, we propose a more dynamic view of InfA as a continuum of related inflammatory states, as opposed to stages. These states are defined by the balance and interaction of regulatory processes that govern the system's behaviour, rather than by absolute concentrations of inflammatory mediators. The inflammatory resolution capacity, the burden of SCs, metabolic and epigenetic feedback and the local tissue microenvironment are among the key modulators. Transitions between states are gradual and context-dependent, which signifies the alteration of the regulatory balance and physiological resilience [16,17]. The state-based view preserves the continuity of earlier inflammaging theories while providing a versatile means of incorporating multidimensional biomarkers, biological clocks and systems-level measurements. Hence, it is more suitable to capture the temporal, tissue-specific and contextual complexity of lifespan InfA, as it allows for a more nuanced understanding of how these factors interact over time and influence overall health outcomes.

In this framework, transitions between inflammatory states are proposed to reflect not simply increasing concentrations of inflammatory mediators, but progressive changes in the balance among inflammatory induction, signal termination, SC generation, immune-mediated clearance, metabolic resilience and tissue repair. We therefore define resolution capacity as an integrated property of the tissue that includes molecular termination of inflammatory signalling together with the recognition and clearance of damaged and SCs by immune surveillance. The local immune context may determine which senescent phenotypes persist, while persistent SCs can reciprocally remodel immune and stromal compartments, generating feedback loops that progressively reduce inflammatory reversibility. This view links InfA to senescence immune surveillance, tissue-specific immune escape and evolutionary trade-offs across the lifespan.

## 2. Molecular drivers of inflammaging

The interplay of a wide range of molecular signalling pathways that control immune activation, cellular responses to stress and metabolic adjustment, and tissue homeostasis leads to chronic, low-grade inflammation in the ageing process. InfA is not a response to one dominant inflammatory mediator but arises from the integrated actions of interdependent signalling networks across immune, metabolic, stress-response and cellular damage pathways [18]. These networks form a coupled regulatory system in which inflammatory activation, metabolic state, mitochondrial dysfunction and feedback regulation influence one another. Some of the most well-investigated InfA drivers include nuclear factor-κB (NF-κB) [19], the Janus kinase-signal transducer and activator of transcription (JAK–STAT) pathway [20], mTOR signalling [21], AMPK [22] and inflammasome activation [23]. Their contribution to InfA therefore depends not only on pathway activation but also on the duration of signalling, reciprocal pathway coupling and the capacity to restore homeostasis.

NF-κB is among the key transcriptional regulators of inflammatory responses and is widely regarded as a principal transcriptional regulator of InfA. NF-κB regulates the expression of many pro-inflammatory genes, including cytokines, chemokines, adhesion molecules and immunosurvival regulators. NF-κB activity is tightly controlled under physiological conditions and becomes transient in response to infection, tissue injury, or metabolic stress [24]. Activation of pattern recognition receptors such as TLRs and NLRs initiates signalling cascades that lead to the degradation of IκB proteins and the nuclear translocation of NF-κB transcription factors [25]. During ageing, DAMPs, SCs, SASP, dysfunctional mitochondria and chronic metabolic stress can promote persistent NF-κB activation and sustained production of TNF-α, IL-6, and IL-1β, contributing to the chronicity of InfA [26,27].

Importantly, the biological consequences of NF-κB signalling depend not only on activation magnitude but also on signal duration and effective termination. Negative regulators, including IκB proteins, A20 (tumor necrosis factor alpha-induced protein 3, TNFAIP3) and phosphatases, normally constrain inflammatory signalling after removal of the initiating stimulus [28]. Progressive impairment of these feedback mechanisms may convert transient NF-κB activity into persistent inflammatory signalling. Within the proposed state-transition framework, failure of signal termination may therefore be more informative than pathway activation alone when distinguishing compensatory from maladaptive or S3 inflammaging.

Cytokine, metabolic and stress-sensing pathways then reinforce this core inflammatory circuitry. Cytokine-dependent JAK–STAT signalling provides a complementary amplification route within this network. The JAK–STAT pathway mediates cytokine signalling that regulates immune function, cellular proliferation and inflammation. Sustained IL-6 and other pro-inflammatory cytokine exposure during ageing chronically activate JAK–STAT signalling, which can reinforce inflammatory transcription and contribute to persistent inflammation. Persistent JAK–STAT activity can reinforce NF-κB-dependent inflammatory programmes and thereby sustain cytokine signalling in ageing tissues [29,30].

Metabolic signalling further couples inflammatory activity to cellular energetic state. The mTOR pathway integrates growth-factor, nutrient, energy and cellular-stress signals to coordinate anabolic and catabolic activities [31]. Sustained mTOR activation in ageing is associated with defective autophagy, metabolic dysfunction and augmented inflammation, and can interact with NF-κB and other inflammatory regulators [32].

In evolutionary terms, the antagonistic–pleiotropy/hyperfunction model can also be applied to persistent mTOR signalling: a signal essential for growth, biosynthesis and tissue development in early life may become detrimental later in life as physiological needs change [21,33]. Persistent mTORC1 activity may favour anabolic and inflammatory programmes at the expense of autophagy, thereby contributing to age-associated dysfunction when it is not appropriately downregulated.

AMPK is a vital cellular energy sensor that helps maintain metabolic homeostasis under energetic stress by activating catabolic pathways in response to high AMP/ATP ratios [34]. Alongside its role in metabolism, AMPK also exerts anti-inflammatory effects by inhibiting the pro-inflammatory NF-κB and mTOR signalling pathways. Age-associated metabolic and mitochondrial dysfunction can reduce AMPK activity, linking the mTOR–AMPK balance to inflammatory persistence and reduced metabolic flexibility [22].

The cGAS–STING pathway provides an important mechanistic link between age-associated genomic or mitochondrial damage and sterile inflammation. Mitochondrial dysfunction, genomic instability and defective organelle clearance can increase the accumulation of cytosolic DNA, including mtDNA. Cytosolic DNA is sensed by cyclic cGAS, leading to cGAMP production and activation of STING, which engages TBK1–IRF3-dependent type I interferon signalling and can reinforce NF-κB-mediated inflammatory programmes [35]. Persistent cGAS–STING activity may therefore connect mitochondrial damage, cellular senescence and chronic inflammatory signalling. Defective mitophagy further increases the availability of mtDNA to cytosolic DNA-sensing machinery, positioning cGAS–STING at the interface between declining cellular quality control and inflammaging.

## 3. Physiological origins of inflammation across the lifespan

Inflammatory activity is a fundamental physiological element of the immune system throughout the lifespan. As outlined in the preceding section, this activity is orchestrated by conserved signalling networks that regulate immune activation, metabolic sensing and stress adaptation. Innate immune components are present during early development, including the in-utero period, where regulated inflammatory signalling contributes to host defense, tissue development, barrier formation and physiological regulation. Early immune activity involves basal signalling and inducible inflammatory responses that are tightly controlled according to developmental and environmental cues, supporting tissue homeostasis [36,37].

With the maturation of immune functions, adaptive immunity develops and combines with the already existing innate inflammatory reactions. Adaptive immune responses are specific and memory-based but need to be activated, controlled and resolved by the innate immune system. In this integrated system, inflammatory responses are beneficial for effective adaptation to stress and recovery of homeostasis. As one ages, there is progressive loss of both innate and adaptive immune regulation, which changes the balance and effectiveness of these responses [38,39]. Together, these observations highlight the fact that inflammation is not a fixed or always harmful process, but rather, it is a process that is constantly remodelled throughout the lifespan. Since immune regulation varies with age and context, the qualitative nature of inflammatory activity changes in a similar fashion, generating a continuum of inflammatory states rather than discrete chronological phases. In this context, inflammaging should be understood not as a late-life phenomenon but as the cumulative outcome of progressive regulatory remodelling across the lifespan.

The theory of antagonistic pleiotropy offers an evolutionary interpretation of this lifespan trajectory: biological traits that confer benefits early in life can impose costs later in life as natural selection weakens after reproduction [33]. Within this framework, inflammatory responses that benefit host defense, tissue repair and developmental remodelling may become maladaptive as regulatory control is progressively challenged later in life. Likewise, SCs may be beneficial early in life by limiting the growth of damaged or potentially cancerous cells. Still, long-lived SCs and their inflammatory products could be harmful later in life, leading to tissue dysfunction and cancer-promoting microenvironments [40].

The proposed S0–S3 framework can thus be considered not only a description of progressive inflammatory dysfunction, but also an evolutionary and physiological consequence of rebalancing biological processes that were originally selected for beneficial functions and are now age-dependent. This framework also helps contextualize the apparent centenarian paradox: the persistence of effective tumour-suppressive and inflammatory regulatory mechanisms in exceptionally long-lived individuals does not contradict antagonistic pleiotropy, because the evolutionary model predicts a distribution of outcomes rather than inevitable late-life deterioration [41]. Individuals or tissues that retain stronger regulatory, repair, immune-clearance and metabolic resilience may remain closer to adaptive inflammatory states despite advanced chronological age.

## 4. A state-based framework for inflammaging

InfA can be conceptualized as a system of interrelated inflammatory states that helps explain the complex and heterogeneous inflammatory changes associated with ageing. These states are not fixed chronological stages or universal paths, but descriptive reference points that indicate shifts in regulatory balance, resolution capacity and system resilience [42]. It is important to note that individual states can co-exist in tissues and may vary depending on the situation. This framework serves as a flexible structure to integrate molecular, cellular and systemic observations of age-related inflammation [43].

In contrast to classical linear or stage-based models of ageing, which imply a uniform and time-dependent progression, this state-based approach emphasizes regulatory dynamics, reversibility and tissue-specific heterogeneity as defining features of InfA. Operationally, resolution capacity in this framework refers to the integrated ability of a tissue to terminate inflammatory signalling, clear damaged cells and SCs, restore metabolic and mitochondrial homeostasis, remove inflammatory debris and re-establish tissue function after stress. It therefore encompasses both molecular termination mechanisms and cellular immune-surveillance mechanisms. A particularly important component is the balance between the rate at which SCs are generated and the capacity of NK cells, CD8^+^ and CD4^+^ T cells and macrophages to recognize and eliminate them [44,45].

Whether SC accumulation remains transient or becomes persistent depends in part on the effectiveness of immune surveillance. *In vivo*, SCs have been demonstrated to be actively recognized and eliminated by context-dependent interactions with innate and adaptive immune cells. In the liver, oncogene-induced senescent hepatocytes undergo immune-mediated clearance involving CD4^+^ T cells and monocyte/macrophage populations. Bycontrast, impaired senescence surveillance promotes persistence of premalignant senescent hepatocytes and liver tumour development [44]. Furthermore, NK cells can kill senescent tumour cells via NKG2D-mediated recognition, and senescence-associated chemokines are involved in NK cell recruitment [46]. Conversely, SCs can acquire immune-evasive phenotypes. SCs express HLA-E, which interacts with the inhibitory receptor NKG2A on NK cells and differentiated CD8^+^ T cells, reducing their cytotoxic surveillance. At the same time, blockade of the HLA-E–NKG2A interaction enhances NK- and CD8^+^ T-cell-mediated recognition and clearance of SCs [47]. More recently, increased expression of the ganglioside GD3 has been shown to impair NK cell surveillance and induce an immune-privileged phenotype in SCs during ageing. By contrast, anti-GD3 immunotherapy has been shown to decrease age-related tissue pathology in experimental models [45]. Together, these findings support the view that resolution capacity depends not only on termination of inflammatory signalling but also on the effectiveness of tissue-specific immune recognition and clearance of SCs, with immune-evasion mechanisms favouring their persistence.

### (a) S0–adaptive/resolved inflammatory state: preserved regulatory homeostasis

Efficient senescence immune surveillance is proposed to be a defining feature of S0. Senescent or damaged cells generated during physiological stress are rapidly recognized and removed by context-dependent innate and adaptive immune mechanisms, preventing their persistent accumulation and limiting prolonged exposure to senescence-associated inflammatory mediators [48]. Thus, SC generation remains balanced by effective clearance, while disease-tolerance mechanisms can preserve tissue function during transient stress. Recent evidence further indicates that p16^High^ immune cells can contribute to disease tolerance by limiting tissue injury during severe inflammatory stress without necessarily reducing the underlying pathogen burden. Triana-Martinez *et al.* [49] showed that induction of p16^High^ immune subsets promoted protection against endotoxin shock, bacterial sepsis and irradiation-associated tissue injury, whereas genetic or pharmacological depletion of these cells abolished the protective effect.

As outlined earlier, inflammation has been a part of a tightly regulated physiological process since the earliest age of life. In the resolved and adaptive state (S0), inflammatory signalling operates as a tightly controlled and reversible process that supports host defense, tissue repair and the maintenance of physiological homeostasis. Innate immune sensing elements, such as NLRs, TLRs and RLR, are activated by inflammatory signals in the S0 state [50]. These receptors activate the downstream signalling pathways in response to endogenous stress signals and metabolites produced by the microbiota [51].

In this inflammatory state, the classical proinflammatory cytokines (TNF-α, IL-1β and IL-6) are released in a regulated and spatially restricted manner. Their main function is to recruit immune cells and orchestrate host defense and tissue homeostasis to contain the insult and coordinate tissue repair [52]. Another characteristic of S0 is the efficiency with which inflammation is resolved. Pro-inflammatory signalling is balanced by anti-inflammatory signalling that is mediated by IL-10, transforming growth factor-beta (TGF-β) and specialized pro-resolving lipid mediators. These mechanisms regulate the duration of exposure to cytokines and prevent collateral damage to the tissues [53]. The process of immune cell turnover is predominated by apoptosis, which is a form of immunologically silent programmed cell death. Phosphatidylserine, an ‘eat me’ signal on the surface of apoptotic cells, further promotes their clearance by macrophages through efferocytosis. This process inhibits the release of DAMPs and secondary immune activation, which can otherwise lead to chronic inflammation and autoimmune responses [54].

The key to the sustainability of S0 is metabolic resilience, which facilitates effective immunity and cell integrity. The redox pathways, mitochondrial oxidative phosphorylation and glycolysis are harmonized to provide the required amount of energy without causing metabolic stress. ROS are produced transiently for antimicrobial protection but are tightly buffered by antioxidant systems [55,56]. In this state, the inflammatory pathways that are activated in a somewhat reversible and controlled manner are the MAPK, NF-κB and interferon regulatory factor (IRF) networks. After the elimination of the inflammatory stimulus, the signalling is terminated by the feedback inhibitors, including IκB and phosphatases [57–59].

Collectively, S0 is characterized by transient cytokine elevation, efficient efferocytosis, preserved mitochondrial function, balanced redox signalling and intact feedback inhibition—features that may correspond to low basal circulating cytokine levels and robust resolution responses *in vivo*. Thus, adaptive or resolved inflammation is inducible and requires appropriate physiological or pathological triggers. Loss of efficiency in any of these regulatory modules—resolution, metabolic buffering or feedback inhibition—may predispose tissues to transition toward more persistent inflammatory states.

### (b) S1-compensatory inflammaging: baseline elevation with preserved control

In the proposed S1 state, the generation of senescent and stressed cells may increase, but immune clearance still largely compensates for this increased burden. NK-cell, T-cell and macrophage surveillance remain sufficiently functional to prevent stable accumulation of highly inflammatory senescent populations. Inflammatory termination and metabolic adaptation are delayed but preserved, consistent with greater reversibility of this compensatory state.

Unlike resolved inflammation in S0, inflammatory activity in S1 is no longer strictly episodic and low levels of pro-inflammatory mediators are chronically detectable in circulation. Population studies have consistently reported modest increases in circulating cytokines such as IL-6, TNF-α and CRP in otherwise healthy older individuals. Importantly, these increases are usually within a two- to four-fold range as compared with younger populations, distinguishing S1 from overt inflammatory disease states [10,60].

Cell death modalities markedly influence inflammatory outcomes: necrotic cell death exposes intracellular contents that function as pro-inflammatory signals, whereas apoptosis typically limits inflammatory signalling by sequestering potential DAMPs for silent clearance. These mechanisms underline how DAMPs released from dying cells activate innate immune receptors and drive inflammatory cascades in sterile injury and disease contexts. More importantly, counter-regulatory mechanisms are still in place [61]. Inflammatory amplification remains constrained by anti-inflammatory mediators, NF-κB negative feedback and resolution pathways. This state is also characterized by metabolic adaptation. Immune and stromal cells can react to chronic exposure to cytokines with adaptive changes in mitochondrial respiration, glycolysis and redox management. Inflammatory signalling is increasingly coupled with metabolic control, allowing cells to survive chronic low-grade stress without an acute loss of functionality. This metabolic plasticity helps preserve cellular resilience despite a persistently elevated inflammatory baseline [62,63].

The conceptual features of S1 are consistent with pre-inflammatory or cold-InfA conditions, which are characterized by a subtle shift in homeostatic balance rather than disruption. In this condition, inflammation is an indication of compensation and not failure. Notably, the inflammatory condition is highly heterogeneous across individuals and tissues and can last for a long time. The shift towards maladaptive InfA is not a predetermined process, and it relies on the quality of regulatory, metabolic and clearance processes [10,64]. S1 represents a buffering phase in which inflammatory baseline displacement is evident, yet regulatory hierarchies remain intact. Plasticity is preserved, and progression toward maladaptive inflammaging depends on the durability of metabolic, clearance and feedback reserves. Thus, in S1, increased SC generation is proposed to remain largely compensated by immune clearance, limiting persistent accumulation despite a chronically elevated inflammatory baseline.

### (c) S2-maladaptive but potentially reversible inflammaging: emergence of coupled dysregulation

A defining feature of maladaptive InfA (S2) is proposed to be a mismatch between increasing SC generation and declining immune-clearance capacity. SCs can increasingly persist through immune-evasion mechanisms, including HLA-E/NKG2A signalling, GD3-associated suppression of NK-cell surveillance and checkpoint pathways such as PD-L1 and CD47 [45,47]. These mechanisms are strongly influenced by tissue and biological context, such that the capacity of immune surveillance to recognize and eliminate SCs varies across tissues and senescence-inducing conditions [48].

Maladaptive InfA (S2) is characterized by constrained metabolic flexibility in immune and stromal cells owing to impaired mitochondrial responsiveness and persistent reliance on glycolytic and biosynthetic programmes. Glucose flux is preferentially redirected toward redox buffering and biosynthetic pathways, including the pentose phosphate pathway and anaplerotic reactions, thereby sustaining cellular survival and cytokine production. This adaptive redox stability occurs at the expense of energetic efficiency and dynamic resilience, reinforcing a persistent low-grade inflammatory tone [65–68].

Inflammatory signalling through NF-κB and inflammasome-associated pathways becomes prolonged rather than occurring as transient pulses. These processes may overlap with telomere dysfunction and telomerase insufficiency, which increase DDRs mitochondrial stress, and inflammatory vulnerability [69,70]. Moreover, because telomerase activity depends on the abundance and assembly of multiple telomerase components, measurements of TERT alone may not fully capture functional telomerase status across cellular contexts [69]. Consequently, inflammatory signalling becomes self-referential and responds to internally generated stress signals rather than external challenges.

Another major contributing factor for persistent inflammatory tone is intercellular communication. Exosomes (extracellular vesicles) facilitate the transfer of stress-related molecular cargo between cells and tissues, promoting the spatial and temporal propagation of inflammatory signals. These exosomes mediate the interaction of inflammatory, metabolic and stress-response signals between cellular compartments by augmenting pre-existing dysregulation rather than new inflammation [71,72].

Experimental studies provide evidence that several biological features relevant to the proposed maladaptive S2 state remain therapeutically modifiable. Disruption of the HLA-E–NKG2A interaction enhances NK- and CD8^+^ T-cell responses against SCs, while targeting GD3 reduces senescence-associated immune evasion and age-related pathological remodelling in experimental models [45,47]. Similarly, anti-uPAR CAR T-cell therapy reduces uPAR-positive senescence-associated cells and improves tissue regeneration, barrier function, inflammation and mucosal immune function in aged mice [73]. Together, these studies indicate that individual features of the proposed S2 state—including impaired immune recognition, SC persistence and associated inflammatory dysfunction—can remain therapeutically modifiable. Thus, S2 is proposed as a potential therapeutic window in which maladaptive inflammatory and cellular-stress processes may remain partially reversible, pending prospective experimental and clinical validation.

### (d) S3-self-sustaining inflammaging: autonomous inflammatory behaviour and loss of termination

Self-sustaining InfA (S3) is characterized by inflammatory activity that may become relatively independent of the initiating external stimulus [74]. Rather, inflammatory signalling becomes an intrinsic property of system behaviour. The hallmark of S3 is the failure of the termination mechanism. Ongoing inflammatory signalling persists because time-limiting termination mechanisms become ineffective, resulting in sustained activation [75]. This stands out as opposed to the previous states, where persistence was conditional and reversible.

Cell-intrinsic stress responses are important for maintaining inflammatory activity [76]. The perpetuation of this inflammatory response is supported by the unremitting production of intracellular signals generated by genomic instability, telomere dysfunction and mitochondrial impairment [77]. Telomerase insufficiency promotes telomere shortening and the subsequent triggering of chronic DDR and redox imbalance. In this regard, inflammatory signals are more sensitive to internal cellular stress than to environmental stimuli. Communication between cells and tissues further stabilizes this condition [78]. These signals coordinate the inflammatory response of neighbouring and distant cells, thus driving short-range propagation of inflammation [79,80]. This spatial continuity occurs in a context where local resolution capacity is impaired because of a defect in efferocytic clearance and specialized pro-resolving mediator pathways [81]. In the presence of persistent injury, inflammation therefore acquires spatio-temporal continuity, leading to immune system structural remodelling, including novel proinflammatory macrophage populations in aged tissues and myeloid clones expanding from clonal haematopoiesis with enhanced inflammatory capacity [80]. These long-lasting cellular changes are strengthened by infection-induced senescence. In the proposed S3 state, inflammatory circuitry may become increasingly embedded at genomic, epigenetic and cellular population levels, rendering the system progressively insensitive to transient regulatory inputs.

This structural encoding of inflammatory circuitry is reinforced by three major biological amplifiers: cellular senescence, trained immunity and clonal haematopoiesis. SCs develop a secretory phenotype, characterized by persistent production of pro-inflammatory cytokines. At the same time, trained immunity triggers epigenetic reprogramming of innate immune cells, creating a state of increased and sustained responsiveness. This reorganization of immune-cell composition and function may sustain non-resolving chronic inflammation. Consequently, inflammatory responses may become less effectively coupled with transient stimuli and resolution. Age-associated clonal haematopoiesis, maladaptive trained immune programmes and accumulation of SCs may each contribute to persistent inflammatory signalling and reduced resolution capacity [79,82].

Immune escape in S3 is therefore proposed to extend beyond individual checkpoint-dependent mechanisms and become embedded within broader defects in immune recognition and effector function. Reduced antigen presentation and persistent checkpoint signalling may limit effective CD8^+^ T-cell recognition and contribute to T-cell dysfunction, while altered myeloid populations may further suppress cytotoxic immune activity [48]. Together, these changes may reinforce the persistent, relatively stimulus-independent inflammatory state proposed for S3.

Operationally, the distinction between S2 and S3 should depend on the breadth and reversibility of regulatory dysfunction rather than inflammatory magnitude alone. S2-like tissues would be expected to retain measurable immune-surveillance and signal-termination capacity despite increased SC burden, whereas S3-like tissues would show persistent SC accumulation together with broader immune remodelling, impaired antigen presentation and lymphocyte function, altered myeloid states and evidence of trained immunity or clonal haematopoiesis [48,79]. Coordinated tissue-level assessment of SC burden, immune surveillance, signal termination, metabolic resilience and immune/clonal remodelling may therefore distinguish S2 from S3 more effectively than circulating inflammatory markers alone. Circulating biomarkers may support screening and longitudinal monitoring, but they should not be used alone to classify tissue-specific S2 or S3 states; tissue, spatial, cellular and functional measurements will be required for operational validation.

The framework generates several testable predictions. First, interventions that restore SC recognition or inflammatory signal termination should produce greater reductions in SC burden and inflammatory output in S2-like than in S3-like tissues. Second, S3-like tissues should show incomplete responses to single-pathway interventions because inflammatory persistence is maintained by multiple reinforcing mechanisms. Third, longitudinal tissue-level measurements should demonstrate greater reversibility of immune, metabolic and inflammatory abnormalities in S2 than in S3.

In summary, S3 represents the most advanced form of InfA within this framework, characterized by constitutive inflammatory signalling, ineffective termination and stimulus independence. Inflammation is proposed to become an increasingly stable system property rather than a reactive or compensatory response. Age-associated pathologies emerge as system-level consequences of sustained inflammatory architecture rather than isolated disease processes.

A conceptual overview of the four InfA transition states is presented in table 1.

**Table 1.** InfA as a state transition and dynamic process.

feature	S0—resolved/adaptive	S1—compensatory	S2—maladaptive (potentially reversible)	S3—self-sustaining	potential measurable indicators	sample/compartment	representative reference
inflammatory role	physiological, protective	adaptive buffering	dysfunctional adaptation	autonomous persistence	tissue inflammatory gene signatures; cytokine/chemokine profiles; tissue inflammatory pathology	tissue	[43]
cytokine behaviour	transient, inducible	baseline-shifted, low-grade	persistent, amplified	constitutive, uncoupled	serial IL-6, TNF-α, IL-1β measurements; inflammatory-response kinetics	blood/plasma or serum; longitudinal	[83]
circulating cytokine pattern/interpretability	near baseline; inducible	modestly elevated; low-grade	persistently elevated; context-dependent	persistent/variable; limited interpretability from circulation alone	plasma/serum IL-6, TNF-α, CRP	plasma/serum	[83]
resolution capacity	rapid and dominant	preserved but delayed	impaired	largely ineffective	signal-decay kinetics; efferocytic activity; SC clearance	tissue/cellular	[74]
termination of signalling	efficient	slowed	incomplete	failed	IκB/A20 (TNFAIP3) expression; phosphatase activity; NF-κB persistence	tissue/cellular	[24]
metabolic state	fully flexible	adaptively biased	constrained responsiveness	chronically dysregulated	AMPK/mTOR activity; glycolytic flux; mitochondrial respiration	tissue/cellular	[34]
mitochondrial function	responsive	stress-adaptive	impaired activation	persistent dysfunction	mitochondrial respiration; membrane potential; ROS; cytosolic mtDNA	tissue/cellular	[26]
cell death balance	predominantly apoptotic	apoptosis largely preserved	mixed cell-death programmes predicted	greater contribution of inflammatory cell-death pathways predicted	apoptosis/caspase activity; markers of inflammatory cell death	tissue/cellular	[84]
intercellular signalling	limited, local	emerging systemic tone	vesicle-mediated reinforcement	cross-tissue propagation	extracellular-vesicle abundance/cargo; cytokine/chemokine exchange	plasma/serum and tissue/cellular	[72]
genomic/telomere stress	minimal	compensated	active but buffered	persistent and destabilizing	γH2AX/DDR markers; telomere length/dysfunction; telomerase activity	tissue/cellular; blood-derived cells where applicable	[85]
immune architecture	balanced	functional	reduced/escape-prone	structurally remodelled; exhausted/ suppressive	NK/CD4^+^/CD8^+^ T-cell abundance/function; HLA-E/NKG2A; GD3; immune-checkpoint markers	tissue/spatial; blood	[48]
spatial pattern	coordinated	mild heterogeneity	increasing divergence	tissue-autonomous	multiplex imaging; spatial transcriptomics; tissue-level immune/SC mapping	tissue/spatial	[48]
proposed plasticity/reversibility	high	high	conditional	low	longitudinal change after intervention; recovery of inflammatory, immune, and metabolic parameters	longitudinal; blood ± tissue	[73]

The references provide representative support for the biological features or candidate measurements listed in each row and should not be interpreted as independent validation of the proposed S0–S3 state assignments. The listed indicators are intended as candidate, tissue- and context-dependent readouts for hypothesis testing, and the S0–S3 framework remains conceptual pending prospective experimental and clinical validation.

## 5. State-dependent senescent modulation of inflammatory microenvironments

Beyond its classical tumour-suppressive function, cellular senescence has emerged as a heterogeneous driver of tissue inflammation. SCs accumulate progressively with age across multiple tissues and acquire distinct secretory phenotypes that influence surrounding cellular environments. Heterogeneity in the inflammatory behaviour of SCs is mediated by distinct molecular programmes, rather than a common SASP. Evidence from diverse tissues demonstrates that SCs adopt distinct functional states shaped by specific transcriptional programmes, metabolic dependencies and immune interactions [86–88]. Distinct senescent states are associated with differential engagement of canonical cell-cycle and stress-response regulators, including CDKN1A (p21CIP1) [89], p16INK4a/CDKN2A [90], TP53 [91] and RB1 [92], which influence both cell-cycle arrest stability and inflammatory output. Consequently, SC burden alone cannot predict tissue dysfunction and inflammatory impact.

Beyond nuclear DNA damage, mitochondrial stress-derived nucleic acids critically shape the inflammatory output of SCs [93]. Mitochondrial RNA release into the cytosol triggers the innate immune receptors RIG-I (DDX58) and MDA5 (IFIH1), which communicate via MAVS to activate interferon-stimulated gene programmes. This pathway promotes inflammatory mediator expression independently of classical SASP transcriptional programmes, thereby linking mitochondrial integrity directly to senescence-associated inflammation. Notably, this process can only be triggered in SCs that have impaired mitochondrial RNA processing or membrane integrity [94], thereby defining a mechanistically distinct inflammatory subpopulation.

Along with inherent molecular and metabolic factors, the extrinsic tissue microenvironment has a profound influence on the inflammatory behaviour of SCs. Senescent inflammatory phenotypes are also further diversified by metabolic reprogramming. Specific senescent subsets rely on sustained glycolysis, supported by enzymes such as PGAM1, alongside checkpoint signalling through CHK1 and transcriptional regulation mediated by factors including HIF-2α (EPAS1) and FOXM1. This reorganization of metabolism aids in the survival of SCs, prevents apoptosis and maintains the inflammatory capacity of SCs [95]. In adipocyte-rich niches, dysregulated lipid handling generates oxidative stress that promotes senescence and inflammatory reprogramming in adjacent immune cells. Fatty acid uptake mediated by FABP4 leads to lipid peroxidation and activation of stress responses in CD8^+^ T cells, resulting in a senescent phenotype with altered cytokine signalling and impaired cytotoxic function [96]. These observations support the concept that tissue-specific metabolic cues shape the inflammatory properties of SCs, contributing to spatial heterogeneity in InfA.

In addition to molecular and microenvironmental determinants, temporal dynamics define distinct inflammatory states of SCs. These cells can persist in a restrained or low-inflammatory state, yet microenvironmental cues can shift this equilibrium toward active inflammatory signalling. This capacity for delayed activation positions SCs as dynamic regulators of tissue inflammation rather than static endpoints [97]. Such temporal plasticity may explain how senescent programmes initiated earlier in life contribute to inflammatory pathologies that emerge later.

The unique element of senescent heterogeneity emerges in the population of immune cells that reorganize the local inflammatory networks by maintaining signalling and changing intercellular communication. Through this persistent signalling, immune senescence links systemic immune ageing with tissue-specific InfA and reinforces organ-specific inflammatory programmes [98]. This type of diversity provides a mechanistic foundation for the asynchronous functional decline that is observed in ageing tissues. This duality also explains the paradox that senescence is tumour-suppressive and later permissive for inflammation [99]. Therapeutically, this diversity has critical implications: indiscriminate activation or targeting of senescence-related pathways may disrupt adaptive inflammatory responses while sparing self-sustaining pathological ones [98].

Through context-dependent inflammatory remodelling, distinct senescent states link cellular stress responses to tissue-specific InfA and ultimately to divergent ageing trajectories [100] (figure 1). Within a state-based model, distinct senescent phenotypes occupy different positions along the spectrum from adaptive and compensatory to maladaptive and self-sustaining inflammation. Their distribution influences how inflammatory states evolve during ageing.

**Figure 1.**
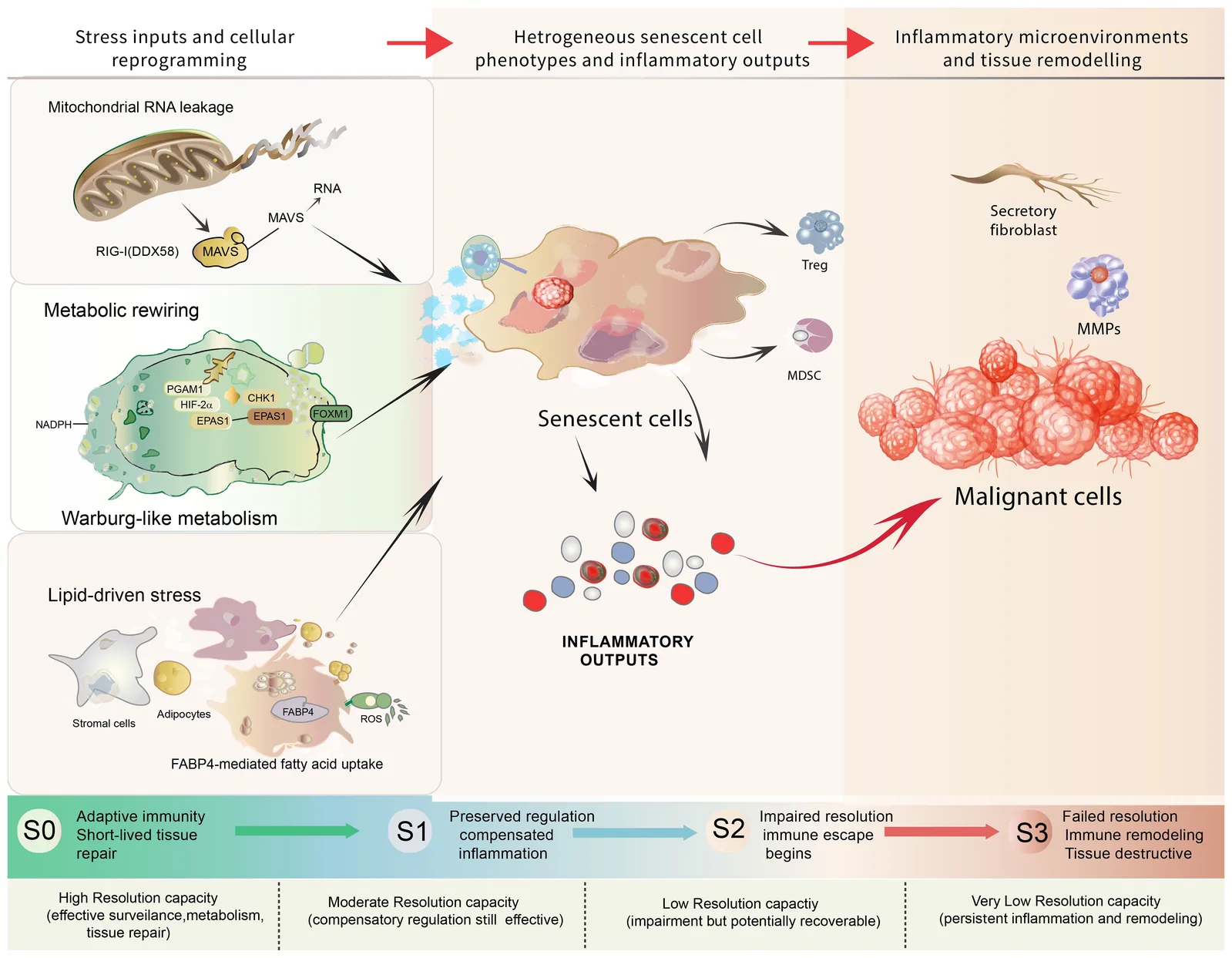
Heterogeneity of SCs modulates inflammatory states and tissue microenvironments during inflammaging. SCs acquire distinct stress-adapted phenotypes according to mitochondrial, metabolic and tissue-specific cues, resulting in heterogeneous inflammatory outputs. These phenotypes interact bidirectionally with immune and stromal compartments, which influence SC recognition and clearance while being reciprocally remodelled by persistent SCs. Across the proposed S0–S3 framework, increasing SC persistence and declining resolution capacity are associated with progressively sustained inflammatory signalling, immune remodelling and tissue dysfunction. S0 represents adaptive, efficiently resolved inflammation; S1 represents compensatory inflammation with preserved regulatory capacity; S2 represents maladaptive inflammation with impaired but potentially recoverable resolution and immune surveillance; and S3 represents self-sustaining inflammation with broader immune and tissue remodelling. The S0–S3 continuum is conceptual and context-dependent rather than a fixed chronological sequence.

Importantly, this heterogeneity can also be shaped by immune surveillance. The local immune context may preferentially eliminate senescent phenotypes that remain immunogenic while permitting persistence of phenotypes with stronger immune-evasive or immunosuppressive properties. Conversely, persistent senescent populations can alter the surrounding immune and stromal compartments through cytokines, chemokines, extracellular vesicles and tissue-remodelling signals, thereby changing the conditions under which subsequent SCs are recognized or cleared [17,72]. This bidirectional selection may therefore contribute to the state-dependent distribution of senescent phenotypes and provide a mechanistic link between SC heterogeneity and progression from compensatory toward maladaptive and S3 InfA.

## 6. Autophagy–inflammation axis in ageing

Autophagy is a natural cellular quality-control process that is very important in cellular homeostasis in ageing. It is an evolutionarily preserved mechanism that facilitates the degradation and recycling of damaged organelles, misfolded proteins and malfunctioning cellular domains via lysosomal degradation pathways [101]. In addition to its classical role, autophagy plays an important role in regulating inflammatory signalling. Autophagy prevents chronic immune activation by eliminating impaired mitochondria, aggregated proteins and intracellular danger signals that activate inflammatory pathways [102].

Autophagic efficiency, however, decreases with age as the organism experiences metabolic stress, mitochondrial malfunction and changes in nutrient-sensing pathways. This deterioration plays a role in the buildup of broken cellular parts that may initiate inflammatory reactions and contribute to the development or persistence of InfA. Mitochondrial quality control is one of the major links between autophagy and inflammatory regulation. Autophagy-based elimination of dysfunctional mitochondria by mitophagy prevents the accumulation of damaged mitochondria that produce excessive ROS and release mtDNA [103].

Mitochondrial damage leads to activation of inflammatory signalling pathways, such as NF-κB activation and inflammasome signalling, when mitophagy is impaired during ageing. Mitochondrial DNA and other mitochondrial components released into the cytosol can act as DAMPs, which trigger innate immune sensors and enhance the amplification of inflammatory signalling. Autophagy also constrains inflammasome activation, especially the NLRP3 inflammasome that is highly associated with age-related inflammatory diseases [104]. In physiological settings, autophagic mechanisms restrain inflammasome stimulation through the elimination of mitochondrial and cytosolic danger signals that cause the production of proinflammatory cytokines. In the case of autophagy impairment, chronic inflammasomes persistently release IL-18 and IL-1β, which are involved in chronic low-grade inflammation. Besides balancing inflammatory signalling, autophagy also regulates cellular senescence pathways. Optimal autophagic activity has the potential to inhibit senescence by keeping mitochondrial activity intact and minimizing oxidative stress. Nevertheless, in cases where senescence has been established, autophagy can counterintuitively preserve the survival of the SCs by supporting their metabolic needs and preserving the SASP [105].

Mechanistically, impaired mitophagy may facilitate state progression by increasing the persistence of damaged mitochondria and the release of mtDNA into the cytosol [106]. Cytosolic mtDNA can activate cGAS–STING–TBK1–IRF3 signalling and type I interferon responses, with additional cross-talk to NF-κB [107]. The mitophagy–mtDNA–cGAS–STING axis therefore provides a direct connection between declining cellular quality control and the transition from compensatory toward maladaptive and potentially S3 inflammation.

## 7. Cancer–ageing paradox: barrier failure, stress adaptation and context-dependent senescence

Cancer is commonly perceived as a disease of old age, with advancing age being one of the strongest risk factors. But tumour-suppressive processes, such as senescence, telomere loss, genome surveillance and immune regulation, also characterize ageing. This phenomenon leads to a basic paradox: biological mechanisms that help prevent tumourigenesis early in life may contribute to tumour development later in life [108] (figure 2). Telomerase illustrates this context-dependent duality: insufficient telomere maintenance contributes to senescence and age-related dysfunction, whereas telomerase reactivation supports continued proliferation in most cancers [70]. To overcome this paradox, it is necessary to go beyond the notion of ageing as a weakening of tumour suppression and to consider how protective mechanisms alter their functional roles with age. Most mutated cells do not develop into cancer. This implies that tumour suppression is active even in old age as a natural evolutionary outcome. Defense mechanisms cannot fully prevent cancer; they can only delay its onset until after reproductive age. The process of ageing is not a failure of tumour control; instead, it changes the mechanism of tumour suppression with time. Genome maintenance pathways demonstrate this transition [112]. High-fidelity DNA repair limits mutational accumulation and maintains the integrity of the cell at an early age. However, as replication stress persists, repair mechanisms become more favourable for the survival of damaged cells instead of their elimination. Genomic instability also has a selective advantage in stressed cells. DNA repair becomes an adaptive rather than protective mechanism in stress, which ensures the persistence of clones [113,114].

**Figure 2.**
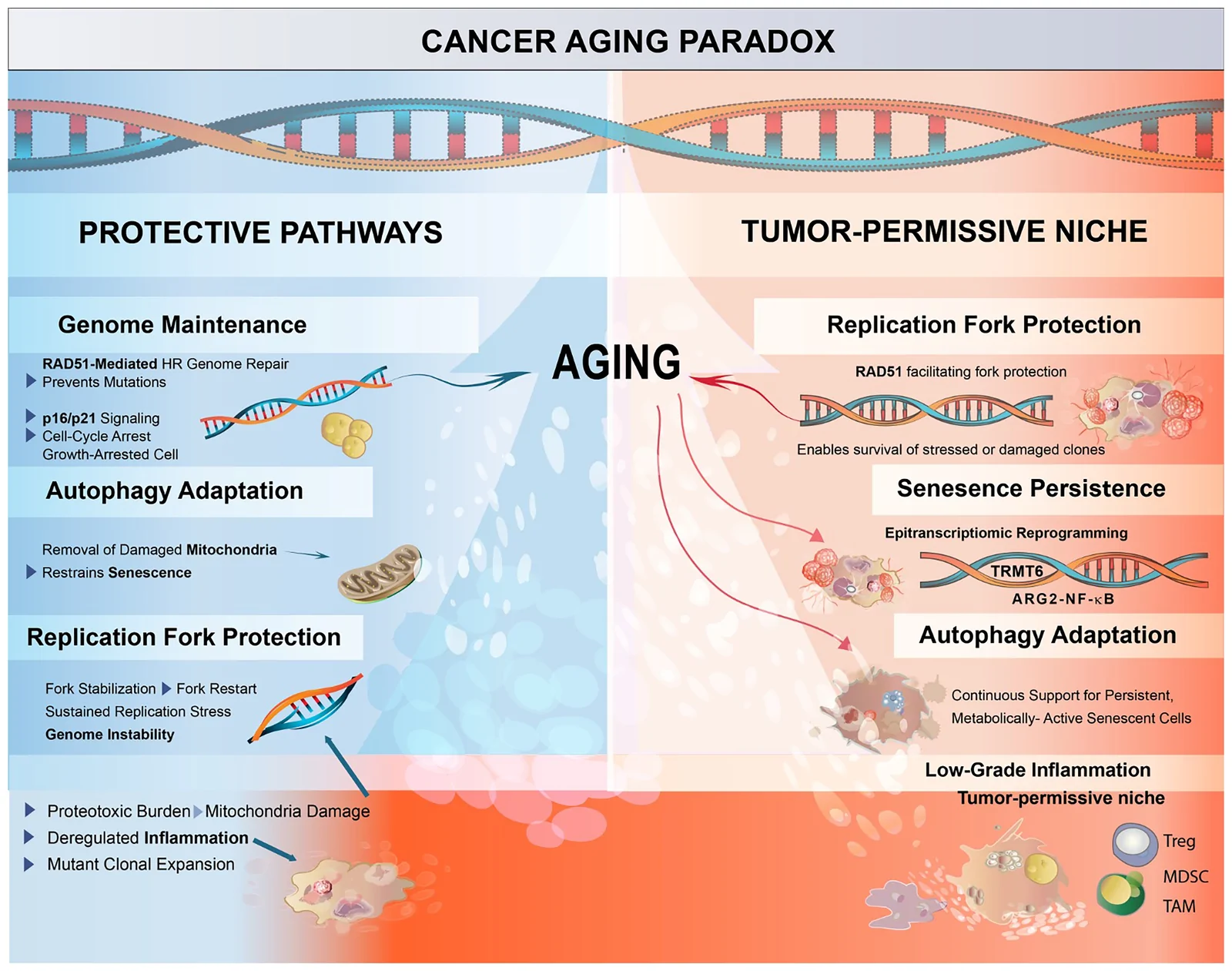
The cancer–ageing paradox: age-associated reprogramming of protective stress-response pathways towards a context-dependent tumour-permissive niche. In younger tissues, genome maintenance, autophagy and replication-fork protection preserve genome integrity, remove damaged cellular components and limit mutant clonal expansion and chronic inflammation. RAD51-mediated homologous recombination facilitates accurate DNA repair, while p16/p21 signalling promotes cell-cycle arrest in damaged cells; autophagy supports mitochondrial quality control and limits proteotoxic stress and replication-fork stabilization and restart help preserve genome stability [109–111]. With ageing, the functional balance of these pathways may become altered, resulting in weakened or rewired protective programmes.

A comparable context-dependent shift occurs in cellular senescence. Initially, senescence acts as a tumour-suppressive mechanism by enforcing durable growth arrest. However, persistent SCs can promote inflammation, stromal activation and immune modulation through their SASP and thus alter the microenvironment of the tissue [115]. A recent study exemplifies that an epitranscriptomic layer in this transition, in which N1-methyladenosine (m1A), a modification catalyzed by TRMT6/TRMT61A, is found to augment the translation of ARG2, thereby stimulating mTOR and NF-κB signalling and promoting a pro-tumourigenic secretory phenotype. This mechanistic connection between microenvironment remodelling and translational control indicates that senescence might favour tumour progression in the face of ageing-related stress [116]. Thus, senescence is not merely a static arrest but a metabolically and epigenetically reprogrammed state.

Thus, the ageing microenvironment is not simply accumulating damage, but is resetting stress-response pathways to tumour-permissive states [117]. ECM remodelling also contributes to the context specificity. One example is the HYAL1-mediated degradation of hyaluronic acid to low molecular weight fragments with pro-angiogenic and immunomodulatory properties, but other studies have indicated that HYAL1 may also play tumour-suppressive roles in some cases. This HYAL1 paradox is a reflection of the larger ageing–cancer paradox: the same pathway can either restrain or promote malignancy, depending upon tissue type, microenvironmental conditions and epigenetic regulation [118].

In aged tissues, persistent replication-fork protection may enable stressed or genomically unstable cells to survive, although this effect is context-dependent and should not be interpreted as uniformly oncogenic. Through epitranscriptomic reprogramming, persistent SCs acquire pro-tumourigenic features, including TRMT6–m¹A signalling and ARG2–NF-κB activation [116]. Metabolically active SCs may adapt through altered autophagy and metabolic programmes that support their persistence and contribute to chronic low-grade inflammation. Subsequently, regulatory T cells, MDSCs and tumour-associated macrophages may contribute to an immunosuppressive microenvironment [119].

## 8. Lifestyle interventions and therapeutic strategies targeting inflammaging and SC heterogeneity

The identification of InfA as an important cause of ARDs has increased research efforts aimed at designing therapeutic approaches to regulate chronic inflammation in ageing. Currently available strategies mainly focus on convergent processes related to chronic inflammation, cellular senescence, immunosenescence and metabolic dysregulation, but not on individual InfA states. These new classes of interventions do not suppress immune responses altogether; instead, they seek to restore homeostasis in inflammatory networks without impairing critical immune processes. Anti-inflammatory, senolytic, senomorphic, immunomodulating and metabolic interventions have demonstrated efficacy, with differing degrees of effectiveness in preclinical models and clinical trials. However, their outcomes are frequently inconsistent, reflecting differences in biological context, disease background and timing of intervention [120–122].

Clinical studies provide emerging but limited evidence for context-dependent effects of ageing-directed interventions. In older adults with diabetic kidney disease, a small open-label pilot study of dasatinib plus quercetin reduced SC burden and selected SASP factors [123]. A randomized trial of TORC1 inhibition in older adults evaluated immune function and infection outcomes [124]. The double-blind MET-PREVENT trial evaluated metformin in older adults with probable sarcopenia and physical prefrailty or frailty, focusing on physical performance [125]. In the CANTOS trial, canakinumab reduced major cardiovascular events in patients with residual inflammatory risk undergoing secondary cardiovascular prevention [126]. Collectively, these studies illustrate context-dependent treatment effects but did not test or validate the proposed S0–S3 states. These studies were conducted in specific disease or at-risk populations and evaluated distinct endpoints; none prospectively classified participants by the proposed S0–S3 framework or tested state-timed therapy.

### (a) Lifestyle and behavioural interventions

Some of the most investigated strategies to regulate ageing-related chronic inflammation are lifestyle interventions such as diet and exercise. Dietary interventions such as caloric restriction and intermittent fasting may reduce inflammatory biomarkers, improve metabolic control and enhance cellular stress tolerance. The activation of metabolic regulators like AMPK and the inhibition of mTOR signalling are among the mediators of these benefits, which promote autophagy and enhance mitochondrial performance [127,128]. Exercise is also an effective anti-inflammatory intervention that enhances metabolic homeostasis, increases mitochondrial productivity and decreases adipose tissue inflammation. It has been demonstrated that exercise can reduce the concentrations of pro-inflammatory cytokines such as IL-6, TNF-α and CRP and increase the release of anti-inflammatory mediators, thus having beneficial effects in chronic inflammatory diseases [129].

### (b) Senolytic and senomorphic therapies

Targeting cellular senescence has emerged as a promising approach for mitigating maladaptive components of InfA. Senolytic drugs induce the selective death of SCs characterized by sustained stress signalling and resistance to programmed cell death. It has been found that senolytic drugs such as dasatinib, quercetin and navitoclax are able to decrease the number of SCs and systemic levels of inflammatory markers in ageing models [130,131]. However, senomorphic therapies are designed to repress the maladaptive secretory phenotype of SCs instead of destroying them. Senomorphic agents can suppress chronic inflammation without disrupting beneficial senescence roles, including tumour-suppressive and tissue-repair functions, by regulating the SASP [132].

Immune-based targeting provides an additional strategy for eliminating senescence-associated cells. uPAR, a senescence-associated surface protein, is increased on cells accumulating in aged tissues, and anti-uPAR CAR T-cell therapy has been shown to reduce uPAR-positive cells and improve tissue regeneration, barrier function, inflammation and mucosal immune function in aged mice [73]. These findings provide proof of concept that senescence-associated surface phenotypes can be therapeutically exploited to enhance immune-mediated clearance.

Importantly, therapeutic strategies should be matched to the dominant biological processes rather than applied uniformly across InfA states. Because inflammation remains adaptive or compensatory in S0 and S1, broad anti-inflammatory therapy may disrupt protective responses and should therefore be reserved for defined pathological indications or elevated disease risk. In S2 and S3, interventions may need to target the specific mechanisms sustaining inflammatory persistence, although such state-matched strategies remain hypothetical pending prospective validation.

### (c) Metabolic and nutrient-sensing interventions

Since metabolic dysregulation is a significant contributor to inflammaging, nutrient-sensing pathway interventions have been of significant interest. Both metabolic activity and inflammatory reactions can be regulated using pharmacological agents that affect AMPK and mTOR signalling [133]. Metformin is one of the most investigated compounds that stimulates AMPK signalling and has been linked to decreased inflammation and improved metabolic health [134]. Rapamycin (an mTOR signalling inhibitor) is another investigated intervention that has been demonstrated to increase lifespan and decrease inflammatory signalling in a number of experimental models [127]. These results suggest a close relationship between metabolic control and inflammatory control during ageing.

These observations support a shift towards state- and mechanism-informed therapeutic strategies. Although there are no strong biomarkers that could consistently identify InfA conditions in humans, the frameworks listed in table 2 provide a conceptual basis to include emerging molecular, metabolic and immune outcomes in the future therapeutic decision-making process. This strategy focuses on adjusting inflammatory mechanisms rather than eliminating them, and the roles of time, context in tissues and biological resilience in intervention development to support healthy ageing.

**Table 2.** Therapeutic strategies targeting inflammaging through SC heterogeneity and inflammatory states.

senescent cell class	dominant mechanism driving inflammaging	key molecular targets	therapeutic class/strategy	representative therapeutic agents (status)	references
stress-sensing SCs (S2–S3)	chronic cellular stress (redox imbalance, mtDNA and chromatin fragments) → NF-κB + type I IFN signalling → chronic SASP production	p53, cGAS–STING, NRF2 balance, mTOR, AMPK, RIG-I, MAVS,	innate immune inhibitors, stress response modulators, senolytics.	FOXO4-DRI peptide*, H-151*, RU.521*, Sulforaphane*, Rapamycin#, Metformin#	[35,128,135–139]
DNA damage-driven SCs (S2–S3)	chronic activation of DDR/ATM/ATR-dependent signalling → p53–p21 / p16 mediated growth arrest → SASP transcription.	ATM, ATR, p53, p21, p16, NF-κB	senolytics, SASP suppression	Dasatinib + Quercetin#, Navitoclax* (toxicity-limited), FOXO4-DRI*	[140–142]
metabolically sustained SCs (S2)	elevated mTORC1 signalling + altered glycolytic and mitochondrial metabolism → maintenance of SC viability and reinforcement of pro-inflammatory secretory programmes	mTORC1, AMPK, NF-κB, SIRT1, PI3K–AKT	gerostatics, metabolic senomorphics	Rapamycin#, Metformin#, Resveratrol*, STACs*, SR1234*	[138,139,142]
microenvironment-programmed SCs (S2–S3)	paracrine TGF-β, IL-1β and IL-6 signalling → reinforcement of senescence and SASP; ECM stiffness may further sustain senescence	TGF-βR, IL-1R, JAK–STAT, NF-κB	cytokine pathway modulation, senomorphics	AlK5 inhibitor (A5i), JAK inhibitors (preclinical in ageing), Rapamycin*	[143,144]
latent SCs (early S2)	impaired immune-mediated clearance of SCs → gradual accumulation of heterogeneous SC populations; sustained activation of SCAPs, including BCL-2/BCL-xL and PI3K–AKT signalling, may promote SC persistence	p21, chromatin modifiers	no validated pharmacologic strategy	no validated pharmacological strategy; candidate biomarkers and targets remain under investigation	[140,145]
immune-evasive SCs (S2–S3)	checkpoint-dependent immune evasion, HLA-E/NKG2A-mediated suppression of NK/CD8^+^ T-cell surveillance and impaired immune-mediated clearance with ageing	PD-1/PD-L1, HLA-E/NKG2A, CD47, uPAR on SCs.	immune senomorphics, immune checkpoint blockade, CAR-T targeting senescence-associated surface markers, immune rejuvenation strategies	anti–PD-1/Anti–PD-L1 antibodies, Monalizumab* (anti-NKG2A), CD47 blockade*,	[45,47,73,146]
broad SC burden (S3)	systemic accumulation of heterogeneous SC populations expressing anti-apoptotic survival networks that sustain chronic low-grade inflammation	CDKN2A, SCAPs BCL-2, BCL-xL, PD-L1, circulating SASP factors	senolytics (apoptosis induction)	Dasatinib + Quercetin# (pilot human), Navitoclax* (toxicity-limited)	[145,147]

*Preclinical evidence; **^#^**clinical evidence

The therapeutic agents listed were not originally developed to target InfA states or senescent heterogeneity. Their relevance to ageing, therefore, depends on timing, tissue context, and dominant inflammatory programmes. Where evidence is indirect or preclinical, this is explicitly indicated.

## 9. Future directions and conclusion

InfA has traditionally been conceptualized as a progressive increase in chronic, low-grade inflammation with advancing age. Although this view has provided an important foundation for understanding age-related inflammatory disorders, it does not fully capture the temporal dynamics, tissue specificity, reversibility and inter-individual heterogeneity of inflammatory remodelling across the lifespan. In this review, we propose that InfA is better understood as a continuum of interconnected inflammatory states rather than a fixed sequence of chronological stages. The proposed S0–S3 framework therefore emphasizes changes in regulatory balance, resolution capacity, metabolic resilience, SC burden, immune surveillance and tissue microenvironment as determinants of inflammatory state and transition. An important implication of this framework is that inflammation and cellular senescence should not be considered intrinsically detrimental processes. Both contribute to physiological functions, including host defense, tissue repair, developmental remodelling and tumour suppression. Their biological consequences depend on the ability of tissues to appropriately terminate inflammatory signalling and eliminate damaged or senescent cells. In this context, the transition from adaptive to pathological InfA may reflect a progressive imbalance between SC generation and immune-mediated clearance. Efficient immune surveillance can restrict the persistence of SCs during adaptive and compensatory states, whereas declining clearance and the emergence of immune-evasion mechanisms may permit specific senescent phenotypes to accumulate and establish self-reinforcing inflammatory microenvironments. This bidirectional interaction between SCs and their immune microenvironment provides a potential mechanistic basis for progression across InfA states.

The framework also provides a broader perspective on the cancer–ageing paradox. Tumour-suppressive mechanisms, including DNA repair, cellular senescence, autophagy and immune surveillance, do not necessarily undergo uniform deterioration with age; rather, their functions may be progressively re-tuned in response to persistent cellular stress, metabolic constraints and changes in the tissue microenvironment. Processes that are protective earlier in life may therefore acquire maladaptive consequences later, consistent with the principles of antagonistic pleiotropy [33]. Cellular senescence, for example, can initially prevent the proliferation of damaged cells, whereas persistent senescent populations and their associated inflammatory and immunomodulatory programs may subsequently facilitate tissue remodelling, immune dysfunction and tumour-permissive microenvironments. Thus, ageing may simultaneously constrain and facilitate malignant evolution depending on the InfA state and tissue context. These considerations have important therapeutic implications. Interventions targeting InfA are unlikely to be uniformly beneficial across all inflammatory states. Suppressing inflammation during adaptive or compensatory responses may interfere with essential host-defense, repair or disease-tolerance mechanisms, whereas interventions during maladaptive states may restore regulatory control and prevent progression towards persistent tissue dysfunction. S2 may therefore represent a proposed therapeutic window because inflammatory dysregulation, metabolic constraints, impaired SC clearance and immune escape are established but may remain conditionally reversible. By contrast, S3 is proposed to involve more structurally embedded changes, including persistent SC populations, immune remodelling, trained immunity, clonal hematopoiesis and impaired inflammatory termination, potentially making restoration of homeostasis substantially more difficult.

A major challenge for future studies will be to determine whether these conceptual InfA states can be operationally distinguished in human tissues. Circulating inflammatory mediators alone are unlikely to adequately capture tissue-specific InfA states, particularly when local immune, stromal, metabolic and senescent-cell programmes become increasingly heterogeneous. Longitudinal studies integrating circulating biomarkers with tissue-level measurements of SC burden and phenotype, immune-surveillance capacity, inflammatory signalling, metabolic and mitochondrial function, epigenetic states and spatial cellular organization will be required to test the proposed framework. Such studies should also establish whether transitions between states are reversible and identify measurable features that distinguish S2 from S3. Collectively, InfA should be viewed as a dynamic balance among inflammatory induction, resolution, SC generation, immune surveillance, metabolic resilience and tissue adaptation. Progressive disruption of these regulatory relationships may drive transitions from adaptive and compensatory states towards maladaptive but potentially reversible inflammation and, ultimately, self-sustaining pathology. Defining tissue-specific biomarkers capable of identifying these states and their transitions will be essential for determining when and how InfA should be therapeutically modulated. Such a state-informed approach may provide a foundation for precision geroscience in which interventions are matched to biological context and timing, with the goal of preserving healthy ageing while reducing age-related disease and cancer risk.

## Data Availability

This article has no additional data.

## Theme

One contribution of 21 to a theme issue ‘Developmental circuits in ageing’.

## Glossary

AMPK, AMP-activated protein kinase; ARDs, age-related diseases; A20, tumor necrosis factor alpha-induced protein 3 (TNFAIP3); CAR T, chimeric antigen receptor T cell; CD47, cluster of differentiation 47; cGAMP, cyclic GMP–AMP; cGAS, cyclic GMP–AMP synthase; CHK, checkpoint kinase 1; CNCD, chronic non-communicable diseases; CRP, C-reactive protein; DAMPs, damage-associated molecular patterns; DDR, DNA damage response; ECM, extracellular matrix; FABP4, fattyacid-binding protein 4; γH2AX, phosphorylated histone H2AX; HYAL1, hyaluronidase-1; FABP4, fatty acid-binding protein 4GD3, disialoganglioside GD3; HLA-E, human leukocyte antigen E; IκB, inhibitory κB; IL, interleukin; IL-1β, interleukin 1 beta; IL-6, interleukin 6; IL-10, interleukin 10; IL-18, interleukin 18; InfA, inflammaging; IRF, interferon regulatory factor family; IRF3, interferon regulatory factor 3; JAK–STAT, Janus kinase–signal ransducer and activator of transcription; m1A, N1-methyladenosine; MAPK, mitogen-activated protein kinase; MAVS, mitochondrial antiviral-signaling protein; MDSC, myeloid-derived suppressor cell; MDSCs, myeloid-derived suppressor cells; MMP, matrixmetalloproteinase; mTOR, mechanistic target of rapamycin; mtDNA, mitochondrial DNA; NF-κB, nuclear factor kappa B; NK, natural killer; NKG2A, natural killer group 2 member A; NLR, nucleotide-binding oligomerization domain (NOD)-like receptor; NLRP3, NOD-like receptor family pyrin domain containing 3; PD-1, programmed cell death protein 1; PD-L1, programmed death-ligand 1; PGAM1, phosphoglycerate mutase 1 ; RIG-I, retinoic acid-inducible gene I; RLR, RIG-I-like receptor; ROS, reactive oxygen species; S0, adaptive/resolved inflammatory state; S1, compensatory inflammaging state; S2, maladaptive inflammaging state; S3, self-sustaining inflammaging state; SASP, senescence-associated secretory phenotype; SC, senescent cell; SCAPs, senescent cell anti-apoptotic pathways; SCs, senescent cells; STING, stimulator of interferon genes; TBK1, TANK-binding kinase 1; TERT, telomerase reverse transcriptase; TGF-β, transforming growth factor beta; TLR, toll-like receptor; TNF-α, tumour necrosis factor alpha; TNFAIP3, tumor necrosis factor alpha-induced protein 3 (A20); TORC1, target of rapamycin complex 1; uPAR, urokinase plasminogen activator receptor.
